# The role of artificial intelligence-based foundation models and “copilots” in cancer pathology: potential and challenges

**DOI:** 10.1186/s13046-025-03592-4

**Published:** 2025-11-28

**Authors:** Cillian H. Cheng, Chi Chun Wong

**Affiliations:** Institute of Digestive Disease and Department of Medicine and Therapeutics, State Key Laboratory of Digestive Disease, Li Ka Shing Institute of Health Sciences, The Chinese University of Hong Kong, Hong Kong SAR, China

**Keywords:** Artificial intelligence (AI), Cancer pathology, Foundation models, Training and validation, AI "copilots"

## Abstract

The integration of Artificial Intelligence (AI) into cancer pathology offers an imperative solution to global pathologist shortages and increasingly complex diagnostic demands. This review summarized the rapid evolution of AI in the field, highlighting the paradigm shift from task-specific (TS) algorithms towards powerful, versatile foundation models (FMs), such as UNI, CONCH, GigaPath, mSTAR, and Atlas. These models, trained on massive and diverse datasets using self-supervised and multimodal learning, demonstrate remarkable capabilities in cancer classification, subtyping, outcome prediction, and biomarker discovery. The emergence of AI "copilots", such as PathChat, SmartPath, further promises to streamline workflows through conversational interfaces and autonomous task planning. However, significant challenges impede clinical translation, including a validation crisis underscored by poor generalizability in zero-shot testing, critical concerns regarding model explainability ("black-box" nature), risks of hallucinations in generative tools, and ensuring generalizability and fairness across diverse populations. Robust external validation, standardized benchmarking, development of explainable AI approaches, and novel regulatory frameworks are essential to responsibly harness the transformative potential of foundation models and realize their promise in improving diagnostic accuracy, efficiency, and patient outcomes in cancer pathology.

## Introduction

Pathology stands as the cornerstone of cancer diagnosis, prognosis, and therapeutic decision-making. The accurate microscopic examination of tissue biopsies by pathologists remains indispensable for determining tumour type, grade, stage, and critical molecular features, directly guiding patient management and treatment pathways. However, this pivotal role is increasingly challenged by a global healthcare landscape straining under significant pressures. For example, a critical shortage of pathologists worldwide, coupled with escalating workloads, threatens diagnostic capacity and timeliness [1, 2]. Simultaneously, the scope of the pathologist’s task has expanded dramatically beyond traditional histomorphological assessment. Modern pathology now demands the integration and interpretation of complex diagnostic techniques, including sophisticated immunohistochemistry (IHC), molecular assays for genetic mutations, and genomic profiling, adding layers of complexity and cognitive burden [3]. These converging pressures, including workforce limitations and escalating diagnostic demands, create an urgent need for innovative solutions to augment pathological practice.

Artificial Intelligence (AI) emerges as a transformative force poised to address these challenges. By leveraging computational power, AI offers the potential to significantly enhance the efficiency, accuracy, and reproducibility of pathological workflows [4, 5]. AI algorithms can automate routine screening tasks, rapidly highlight regions of interest (ROI) in complex slides, reduce diagnostic variability, and uncover subtle, prognostically relevant patterns within tissue architecture that might elude even the trained human eye [6]. Beyond assisting established diagnostic paradigms, AI holds immense promise for discovering novel, previously unrecognized biomarkers by mining the vast, rich data inherent in digitized cancer pathology images and correlating them with molecular and clinical datasets [7]. The feasibility of AI in cancer pathology has been fundamentally enabled by the ongoing digital transformation of the field, primarily through the widespread adoption of whole slide imaging (WSI) [8]. This technology converts physical glass slides into high-resolution digital files, creating the essential data substrate upon which AI algorithms operate [9]. The digitization of cancer pathology archives unlocks the potential for large-scale computational analysis and paves the way for integrating AI tools into diagnostic workflows.

This review aims to systematically explore the rapidly evolving landscape of AI, with a specific focus on the revolutionary impact of foundation models (FMs) and “copilots” within the field of cancer pathology. We will examine the current state of applications, explore key technological advances, critically analyze the persistent challenges in validation and clinical integration, and discuss the future trajectory necessary to realize the full potential of AI as an indispensable tool in improving diagnosis and patient care.

## Highlights of AI's impact on pathology

### Enhanced image segmentation and analysis

A core application of AI in cancer pathology is its ability to precisely segment and classify histopathological images. Deep learning (DL) models are extensively employed in cancer pathology to delineate tumour margins, differentiate malignant from benign tissues, and grade tumours [10, 11]. Coudray et al. [12] trained a deep convolutional neural network (inception v3) on WSIs acquired from The Cancer Genome Atlas (TCGA) to precisely and automatically categorize them into lung adenocarcinoma (LUAD), lung squamous cell carcinoma (LUSC), or normal lung tissue. The performance parallels that of experienced pathologists, attaining an average area under the curve (AUC) of 0.97. Furthermore, AI algorithms significantly improve the quality of low-resolution images [13, 14]. Rong and colleagues [15] developed the Restore-Generative Adversarial Network (GAN), a DL model designed to enhance imaging quality by restoring blurred regions, improving low resolution, and normalizing staining colors.

### Improved diagnostic efficiency and precision

Traditional pathology diagnostics involve manual examination of numerous tissue samples, prolonging cancer diagnosis turnaround times. AI systems rapidly analyze vast quantities of images, substantially accelerating the diagnostic process [16]. Moreover, pathologist interpretation is susceptible to factors such as fatigue, experience level, and sample complexity. AI algorithms can detect subtle alterations in cell morphology, distribution patterns, and other histopathological features that might elude timely human detection, enhancing diagnostic accuracy [3]. Notably, AI has demonstrated significant potential in enhancing the reproducibility of cancer pathology diagnostics. This potential is exemplified by applications including AI-assisted mitosis counting [17], Gleason grading of prostate cancer [18, 19], breast cancer grading [20], counting Ki-67-positive cells [21], and the evaluation of therapeutic targets such as programmed cell death ligand 1 (PD-L1) [22].

### Integration of multimodal data for comprehensive assessment

Moving beyond reliance solely on histopathological images, AI systems increasingly integrate diverse patient data, including genomic sequences, clinical histories, and radiological images, thereby enabling a more comprehensive evaluation [23]. This integrative approach, particularly linking tissue morphological phenotypes with genomic information, is a major research focus. For instance, Jaume et al. [24] utilized complementary data from gene expression profiles to guide slide representation learning through multimodal pre-training. These expression profiles comprise highly detailed molecular descriptions of tissues, which they hypothesize provide a robust, task-independent training signal for the development of slide embeddings. Moreover, research has been conducted to predict gene mutations and protein expression levels directly from cancer pathology images to reduce delays in initiating patient treatment [25].

### Facilitating remote expertise and collaboration

Digitized WSIs can be readily shared globally, enabling remote consultations and collaborative diagnosis [26]. This capability is crucial for accessing specialized expertise. For example, a pathologist in a rural setting can consult an expert at a tertiary medical center, ensuring patients receive high-standard care irrespective of geographic location. Such connectivity promotes collaborative, patient-centered healthcare [27]. A randomized, prospective study conducted by Hanna et al. [28] validated the remote deployment of a digital pathology system, demonstrating operational feasibility for remote review and reporting of pathology specimens, and evaluating remote access performance and usability for remote sign-out. The research achieved major diagnostic equivalence of 100% between digital and glass slide diagnoses, with an overall concordance rate of 98.8% (251 out of 254 cases).

## AI applications in a specific type of cancer

### Colorectal cancer

Based on hematoxylin and eosin staining (H&E)-stained tissue slides, pathologists not only diagnose primary or metastatic colorectal cancer (CRC), including lymph node and distant metastases, but also evaluate various histological features to understand tumour biology. These features include depth of submucosal invasion and microsatellite instability (MSI), which play a role in guiding treatment decisions. Song et al. [29] developed an AI model using a two-step attention-based DL approach without clinical features (AUC, 0.764). In patients with submucosal invasion depth of 1,000 to 2,000 μm, the AI avoided 16.1% of unnecessary additional surgery than using the Japanese Society for Cancer of the Colon and Rectum (JSCCR) guidelines. In addition, automated systems have been created to detect MSI from standard histology slides, offering a more cost-effective alternative to molecular testing. For example, Qiu et al. [30] demonstrated that MSI status can be predicted from H&E-stained images using a DL framework, achieving a cross-validated AUC of 0.809. Notably, fusion models that combined H&E morphology with a single type of molecule yielded superior accuracy, achieving an AUC of 0.952 when H&E images were integrated with DNA methylation data. Another time-consuming and repetitive task in CRC pathology is the microscopic examination of lymph node metastases, particularly given the increased number of lymph nodes harvested through improved surgical and pathological techniques. Khan et al. [31] proposed a DL-based workflow for the detection of lymph node metastases in CRC from H&E-stained WSIs. The methodology demonstrated high sensitivity (0.995, 1.0) and specificity (0.967, 1.0) across two validation cohorts comprising adenocarcinoma cases (*n* = 3,836 slides), by comparing slide-level labels with reports provided by pathologists. Moreover, it has already been demonstrated that AI-assisted workflows enhance the sensitivity of micrometastasis detection (81.94% to 95.83%, *P* < 0.001) and isolated tumour cell identification (67.95% to 96.15%, *P* < 0.001), while substantially reducing review time by 31.5% (*P* < 0.001) [32].

### Breast cancer

Artificial intelligence has been widely applied in breast cancer (BC) diagnosis, primarily focusing on identifying metastatic lesions in sentinel lymph nodes. Since treatment decisions in BC often depend on the detection of these lesions [33, 34]. A key driving force in this area has been the cancer metastases in lymph nodes challenge (CAMELYON), an open competition held in 2016 (CAMELYON16) and 2017 (CAMELYON17) [35]. This AI challenge served as an early model for many cancer-related AI initiatives, providing a publicly available, expertly curated dataset of images along with a transparent framework for evaluating and comparing AI models. In addition, Challa et al. [36] developed a digital imaging analysis tool, which employs the Visiopharm Integrator System (VIS) metastasis AI algorithm to screen lymph nodes for metastases in BC patients, achieving a sensitivity of 100%, specificity of 41.5%, positive predictive value of 29.5%, and negative predictive value of 100%. Instances of false positivity were attributed to histiocytes, crushed lymphocytes, and other factors, which were easily identified during pathologists’ reviews. The aim of this approach is to improve diagnostic accuracy and increase the efficiency of pathologists. Furthermore, AI has been utilized to assist in the diagnosis and classification of BC. For example, Sandbank et al. [37] developed a DL model capable of classifying invasive and non-invasive BC subtypes and predicting clinical and morphological features, which was validated using external datasets from the Institut Curie. Specifically, the areas under the receiver operating characteristic curve (AUROC) for the detection of invasive carcinoma and ductal carcinoma in situ (DCIS) are 0.99 (with a specificity of 93.57% and sensitivity of 95.51%) and 0.98 (with a specificity of 93.79% and sensitivity of 93.20%), respectively [37]. Wang et al. [20] introduced DeepGrade, a DL model aimed at improving the traditional Nottingham grading system for evaluating BC aggressiveness, a framework that has largely been incorporated into Stratipath, a Swedish AI startup specializing in digital pathology.

### Lung cancer

In terms of histological classification, distinguishing lung squamous cell carcinoma from lung adenocarcinoma is usually straightforward. However, the task becomes more challenging when biopsy material is limited or the tumour exhibits poor differentiation. Impressively, Coudray et al. [12] developed a DL model trained on TCGA H&E images to classify non-small-cell lung cancer (NSCLC) subtypes, and their study was also among the first to predict the mutation status of key driver genes directly from H&E images. They demonstrated that STK11, EGFR, FAT1, SETBP1, KRAS and TP53 can be predicted from pathology images, with AUCs ranging from 0.733 to 0.856 on a held-out population [12]. These models are likely to be most beneficial in morphologically challenging cases, making further research into small-cell and large-cell neuroendocrine carcinomas an important area of focus. Histological grading plays a crucial role in prognostication. Pan et al. [38] developed ANORAK, a pyramid pooling cross-stream attention network that encodes multiresolution inputs with an attention mechanism to delineate growth patterns from H&E-stained slides. AI-based grading demonstrated significant prognostic value for disease-free survival and consistently enhanced prognostication for stage I lung adenocarcinomas in a study of 1,372 cases across four independent cohorts, thereby further supporting pathologists in clinical decision-making. Additionally, PD-L1 IHC is routinely performed in all NSCLC cases but is an imperfect biomarker for immunotherapy response, with a weighted AUC of only 0.65 reported in a meta-analysis [39]. This predictive uncertainty probably reflects multiple sources of variance, including tumour heterogeneity and inconsistency in PD-L1 assay performance and subsequent scoring by pathologists. Recent studies have successfully validated automated PD-L1 reading using AI, with multiple models leading to improved interobserver concordance among pathologists [40, 41]. Specifically, without AI assistance, pathologists concordantly classified PD-L1 tumour proportion score in 81.4% of the cases, whereas the overall concordance rate among the pathologists was increased to 90.2% with AI assistance (*P* < 0.001) [40].

## Evolution of AI in pathology: from task-specific tools to foundation models

**Fig. 1:**
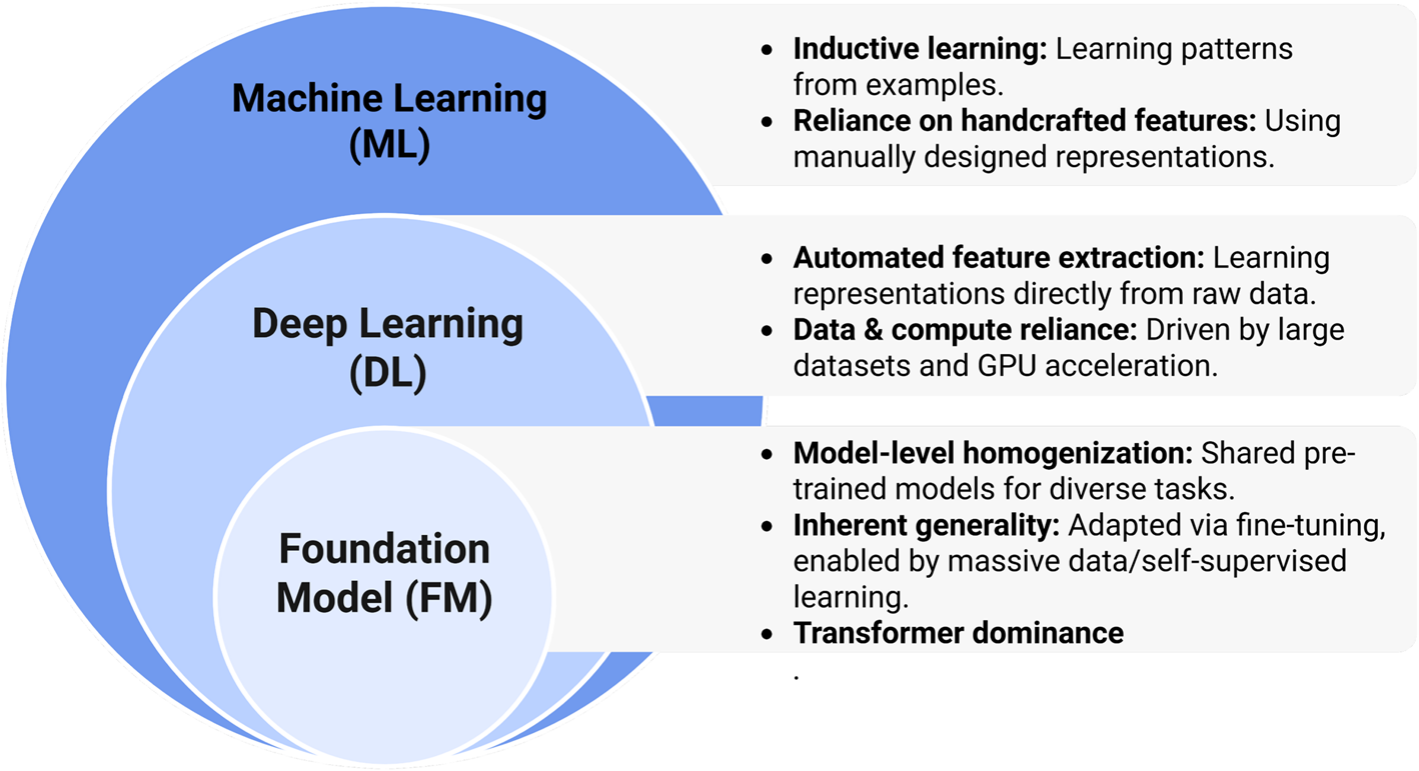
Three prominent stages in the evolution of artificial intelligence: from traditional machine learning, through deep learning, to foundation models

Building on the impact of AI in cancer pathology, the field’s evolution over three decades has progressed through three defining phases: traditional machine learning (ML), DL, and FMs (Fig. 1) [42]. This trajectory marks a paradigm shift from task-specific (TS) algorithms toward versatile FMs capable of addressing core limitations of early systems—particularly in cancer pathology. Crucially, FMs unlock unprecedented capabilities for multimodal data integration, few-shot learning, and clinical workflow augmentation [43].

### The era of task-specific AI: precision with constraints

Early AI applications in pathology (pre-2020s) focused on solving discrete, well-defined problems using supervised learning. These systems excelled in targeted applications but faced fundamental scalability and generalizability constraints [44, 45]. For example, the technical approach primarily relied on convolutional neural networks (CNNs), with architectures such as residual network (ResNet), U-Net, and Inception serving as backbones for feature extraction [46, 47]. Training these models required extensive labeling, including pixel-level annotations (e.g., tumour boundaries) and slide-level labels (e.g., cancer subtypes), which demanded substantial time from pathologists [48–50]. Validation efforts were typically optimized for data from a single institution. However, performance often decreased when applied to external datasets, primarily due to variations in staining protocols or scanner differences [51].

In the field of cancer diagnosis, early AI has demonstrated significant applications but also faces notable limitations. For example, in BC, human epidermal growth factor receptor 2 (HER2) scoring AI effectively reduced inter-observer variability in IHC analysis, yet it struggled to generalize to rare subtypes without retraining [7, 35, 52]. Similarly, in prostate cancer, Gleason grading systems demonstrated higher accuracy compared to average pathologists, achieving a score of approximately 0.70 versus 0.61 [53]. However, they faced challenges when evaluating borderline patterns, such as Gleason 6. Additionally, these systems had limitations, including the need for separate models to analyze biopsies and resection specimens, which consequently increased development costs [54]. In lung cancer, classifiers for NSCLC and small cell lung carcinoma (SCLC) reached an accuracy of 92%, but they were unable to predict molecular markers such as EGFR without dedicated retraining [55]. Beyond these specific applications, several critical shortcomings hinder broader implementation. For instance, one of the major challenges faced by current models is the annotation burden, as developing prostate cancer models required annotating over 112 million patches, which highlights significant scalability barriers [53]. Furthermore, data silos also pose a problem, as models trained on datasets such as TCGA often faltered when applied to slides from community hospitals due to distribution shifts [56]. Consequently, addressing these issues is essential for advancing the robustness and versatility of AI models in cancer pathology.

### The foundation model paradigm shift: a new AI philosophy

Foundation models represent a fundamental rethinking of pathological AI, transitioning from "single-task, single-data" systems to versatile platforms trained on massive, diverse datasets using self-supervised learning (SSL) [57–59]. In recent years, several factors have collectively driven breakthroughs in AI, particularly in medical imaging and pathology. First, the success of generative AI, exemplified by Chat Generative Pre-trained Transformer (ChatGPT), demonstrated that large pretrained models could solve unseen tasks through prompting and fine-tuning, thus expanding the scope of model generalization [60, 61]. From early pioneers such as CTransPath and Lunit in 2022 to recent giants such as CONCH and GigaPath, each boasting over a billion parameters, the field has experienced exponential growth in both model complexity and the scale of training data [62]. Simultaneously, Meta’s release of the DINOv2 vision FM in 2023 proved that the diversity of training data outweighed sheer volume, a principle that has been directly applied to pathology [63, 64]. Additionally, advances in hardware, such as graphics processing unit (GPU) clusters, have enabled training on more than 100 million image tiles, a feat previously limited by computational constraints [65]. At the core of these innovations are self-supervised pretraining techniques, where models learn visual semantics by predicting masked regions or clustering similar features without labels, and transformer architectures, which replaced traditional CNNs with attention mechanisms capable of capturing long-range dependencies across gigapixel WSIs [66–68].

### Landscape of pathology foundation models: architectures and capabilities

**Fig. 2:**
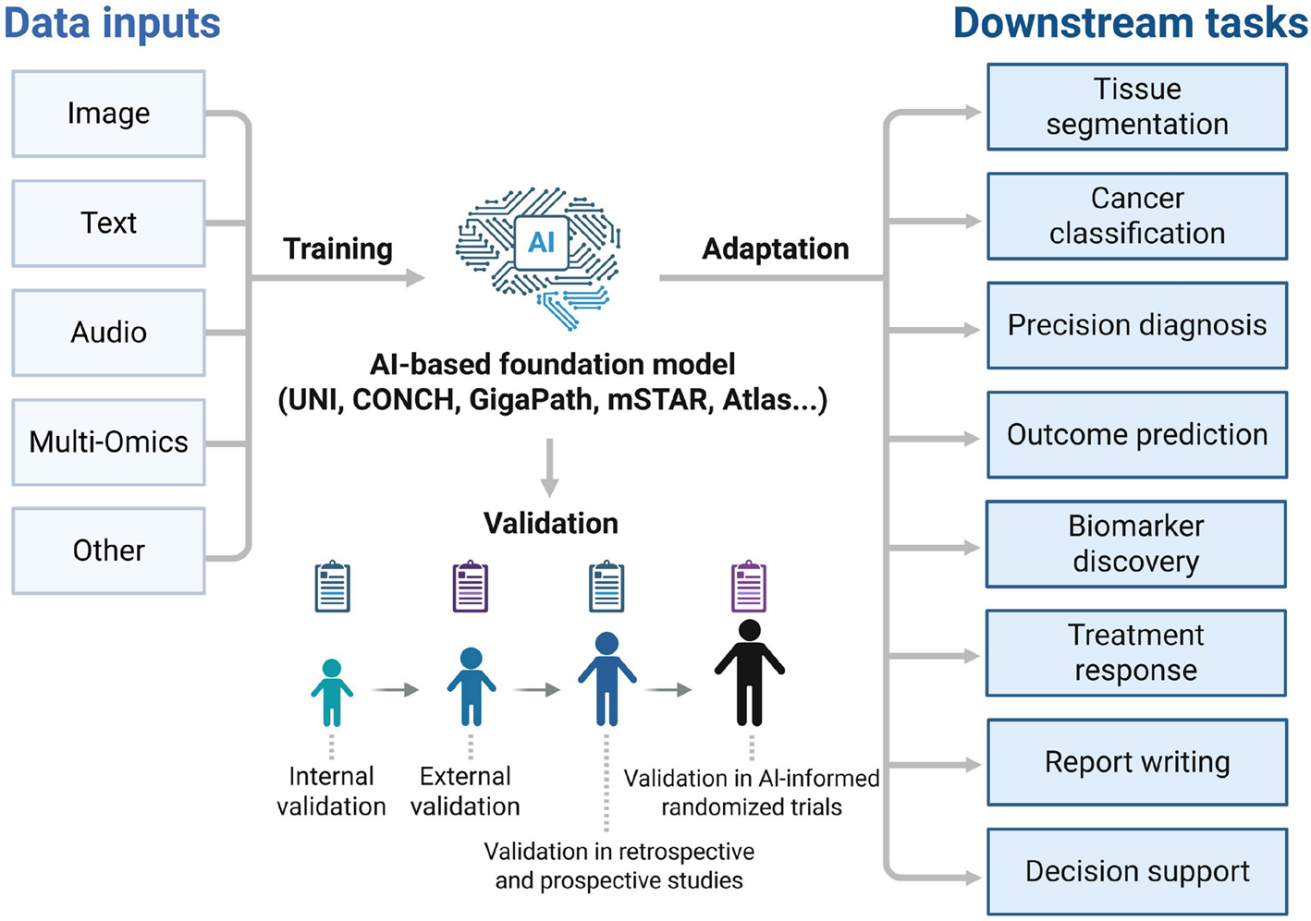
Expanding the use of foundation models to various applications within the field of pathology

The rapid advancement in AI has yielded several powerful FMs for computational pathology (CPath), each with unique architectural innovations, training data, and capabilities (Fig. 2). These models, including UNI, CONCH, GigaPath, mSTAR, and Atlas, signify a paradigm shift toward generalist AI in cancer pathology (Table 1).

**Table 1:** Comparative summary of representative pathology foundation models

Model	Institution/Year	Pretraining Data (Number of Sections/Tissue Type/Multimodality)	Architecture Highlights	Representative Tasks (External Testing)	Applicable Staining	Availability (Open Source/Weight)	Typical Limitations
UNI/UNI2	Mahmood's Lab/2024	• > 100 million images from > 100,000 diagnostic WSIs (Mass-100 K dataset)/ • 20 major tissue types (e.g., breast, lung, brain, kidney, etc.)/ • Unimodal (H&E histology images only)	• Vision Transformer (ViT-Large/16) • Self-supervised learning with DINOv2 algorithm • Combines self-distillation and masked image modeling objectives	• Non-small cell lung cancer (NSCLC) subtyping (CPTAC) • Renal cell carcinoma (RCC) subtyping (CPTAC, DHMC) • Brain tumor subtyping (EBRAINS) • Breast metastasis detection (CAMELYON16) • CRC polyp classification (UniToPatho)	H&E	Yes (Code and model weights available on GitHub for academic research)	• High computational resource demands for pretraining and inference • Unimodal (does not integrate other data types like genomics) • Performance may be influenced by site-specific staining variability • Lacks vision-specific architectural biases for some dense prediction tasks
CONCH	Mahmood's Lab/2024	• 1.17 million histopathology image-caption pairs (after filtering for human tissues)/ • Diverse human tissues/cancers (e.g., breast, lung, kidney, brain, prostate, colon)/ • Multimodal (Histopathology images paired with biomedical text captions)	• Based on the CoCa framework • Image encoder: Vision Transformer (ViT-Base) • Text encoder & Multimodal Decoder: GPT-style models • Training objectives: Combination of contrastive loss (image-text alignment) and captioning loss (generative) • Employed domain-specific unimodal pretraining for both vision and language encoders	Evaluated on 14 diverse benchmarks across multiple external datasets (e.g., TCGA, DHMC, SICAP, DigestPath). Key zero-shot tasks include: • Classification: Breast, lung, and renal cancer subtyping from WSIs; colorectal and prostate tissue classification from image tiles • Cross-modal retrieval: Image-to-text and text-to-image retrieval • Segmentation: Tumor vs. normal tissue segmentation on WSIs	Primarily demonstrated on H&E-stained images. The pretraining dataset also included other stains such as IHC, but the model's performance on non-H&E stains is not the main focus of the evaluation	Yes • Model weights are available for academic research on Hugging Face • Code for using the pretrained model is provided on GitHub	• Scale: Pretraining data scale is smaller compared to billion-scale models in general computer vision • Rare diseases: Struggles with zero-shot recognition of rare diseases in a large class set (e.g., 30-class brain tumor subtyping) • Robustness: Robustness across different staining variations, tissue preparation protocols, and scanners was not fully investigated • Fine-grained concepts: Not designed for cellular or subcellular level tasks (e.g., mitosis detection)
GigaPath	Microsoft/2024	• 171,189 whole slides/ • 31 major tissue types from over 30,000 patients/ • Primarily H&E and immunohistochemistry slides; also explored vision-language with pathology reports	• Novel GigaPath vision transformer architecture • Two-stage pretraining: DINOv2 for tile-level features, then Masked Autoencoder with LongNet for slide-level context • LongNet enables efficient modeling of ultra-long sequences (tens of thousands of tiles per slide)	• Cancer subtyping (9 tasks): e.g., Non-small cell lung cancer, Breast cancer, Renal cell carcinoma • Pathomics (17 tasks): e.g., Pan-cancer 18-biomarker prediction, Lung adenocarcinoma (LUAD) 5-gene mutation (EGFR, KRAS, etc.), Tumor Mutational Burden (TMB) prediction • External test: Achieved state-of-the-art on 25 out of 26 tasks, including on TCGA data	H&E and IHC	Open-weight (Model weights and source code available)	• Performance varies across tasks, especially challenging for some mutation predictions • High computational resource requirements for pretraining • Real-world clinical data not publicly available, limiting full reproducibility
mSTAR	Chen's Lab/2024	• 26,169 slide-level modality pairs from 10,275 patients/ • 32 cancer types from TCGA (e.g., BRCA, LUAD, UCEC)/ • Tri-modal: WSIs, Pathology Reports, and Gene Expression (RNA-Seq) data. (~ 116 million patches)	Two-stage paradigm: • Stage 1 (Slide-level): Trains a slide aggregator (TransMIL) using inter-modality (WSI-Report-Gene) and inter-cancer contrastive learning • Stage 2 (Patch-level): "Self-Taught Training" injects the multimodal knowledge from the aggregator into a ViT-L patch encoder	Evaluated on 15 types of 97 tasks across 7 categories. External testing was performed on datasets from independent medical institutions Representative external tasks: • Pathological subtyping (EBrains, NFGC, YN cohorts) • Metastasis detection (QFS Metastatic) • Mutation prediction (CPTAC cohorts) • IHC biomarker prediction (ZJ1 cohorts) • Molecular subtyping (HANCOCK HPV, ZJ1 Breast) • Survival prediction (OS_HANCOCK, OS_ZJ1) • Report generation (Nanfang, ZJ-First)	H&E	Yes (Code and weights available on GitHub: https://github.com/Innse/mSTAR)	•. Limited paired multimodal data: The scale of pretraining data is naturally limited by the challenge of collecting paired multimodal data, compared to some vision-only foundation models • Not fully end-to-end: The pretraining is not a single, seamless end-to-end process on raw slide data due to hardware limitations, relying on a two-stage workflow as an alternative • Architectural constraint: The slide aggregator (TransMIL) was chosen for linear time complexity, potentially sacrificing some performance for efficiency • Rare cancer assessment: Further assessment of zero-shot performance on real rare cancer cases is needed, though zero-shot tasks on limited data provide some insight
Atlas	Mayo Clinic, Charité, Aignostics/2025	• 1.2 million histopathology WSIs from over 490,000 cases/ • Over 70 tissue/organ types/ • Vision-only model trained on image tiles extracted from WSIs	• Based on the RudolfV training paradigm • Backbone: ViT-H/14 • Parameters: 632 million • Training: Self-supervised learning (DINOv2 framework) on ~ 520 million image tiles at multiple resolutions (0.25, 0.5, 1.0, 2.0 microns per pixel)	Achieved state-of-the-art performance on 11 out of 21 public benchmarks, including: • Morphology-related: BACH (Breast cancer classification), CRC-100 k (Tissue classification), PCAM (Metastasis detection), CAMELYON16 (Metastasis detection), PANDA (Tumor grading) • Molecular-related: MSI STAD (patch) (Microsatellite instability prediction), HEST-PAAD (Gene expression prediction)	Over 100 different staining types, including: • H&E • IHC • Special stains	The report does not state that the model is open source. A related commercial website (Aignostics) mentions "flexible licensing options". Therefore, it is likely not open source and access to the model weights would require a licensing agreement	• Below-average performance on a specific benchmark: Showed lower performance on the TCGA Uniform (20x) cancer subtyping task • Dependence on benchmark diversity: The authors note that the field would benefit from a larger and more diverse pool of public benchmarks to better understand model limitations • Tile-based model: As a tile-based model, its primary representation is for image patches, and slide-level analysis requires an additional aggregation mechanism
RudolfV	Aignostics, TU Berlin, BIFOLD, et al./2024	• 133,998 slides from 34,103 cases/ • 58 tissue types across 14 organ systems/ • Primarily histopathological images; incorporates multiple staining modalities as a key feature	• Base architecture: ViT-L/14 • Training framework: Self-supervised learning with DINOv2 • Key design: Features "pathologist-in-the-loop" curation. This includes pathologist-guided slide grouping (31 groups) and tissue patch clustering (9 clusters) for optimized data sampling, and stain-specific augmentations	• Tumor microenvironment (TME) characterization: Cell classification and tissue segmentation in H&E, evaluated across 5 indications and 8 organs • IHC biomarker scoring: Cell type classification and membrane positivity scoring for markers such as PD-L1, evaluated across multiple cancer types • Reference case search: Retrieval of histologically similar cases for rare diseases from a database of over 6,400 slides • Public benchmarks: State-of-the-art performance reported on PCAM, MHIST, CRC-100 K, and molecular prediction tasks (e.g., MSI)	H&E, IHC (ER, PR, PD-L1, HER-2), and other special stains (Giemsa, PAS, Gomori)	Not explicitly stated in the paper. The model is likely proprietary, as it was developed by a consortium including a commercial entity (Aignostics). No mention of public release of weights or code in the provided text	• Lacks cytopathology and hematopathology cases in its training data • The study does not explore the scaling effects of even larger datasets and model sizes • Utilizes a single pretraining framework (DINOv2) and backbone (ViT); performance of other architectures is unexplored • Does not integrate other data modalities like genomic data or clinical text

*Abbreviations: H&E* hematoxylin and eosin, *IHC* immunohistochemistry, *WSI* whole slide imaging

#### UNI

The UNI/UNI2 models developed by Mahmood’s lab utilize a large vision transformer (ViT-L) architecture coupled with a SSL algorithm DINOv2 pipeline, trained on 200 million image tiles from over 350,000 slides representing more than 20 tissue types [69, 70]. These models introduce key innovations such as scale-agnostic processing, which maintains performance across both biopsy fragments and entire organs, and few-shot adaptation [71]. UNI demonstrates exceptional performance in classifying rare and underrepresented diseases, such as 90 rare cancers within the 108-class OncoTree code classification and various uncommon brain tumours. Chen et al. [58] demonstrated that UNI achieves the highest overall balanced accuracy of 65.7% and AUROC of 0.975 in 32-class pan-cancer tissue classification (19 out of 32 of which are rare cancers), outperforming the next best-performing model by 4.7 percentage points in accuracy and 0.017 in AUROC (both *P* < 0.001). It exhibits remarkable label efficiency, surpassing TS multiple-instance learning methods while using only four slides per class. Additionally, it provides competitive "out-of-the-box" performance on 34 evaluated tasks compared to specialized models, advocating for generalist AI approaches over narrow TS models in CPath. However, limitations include reduced performance in dense prediction tasks such as segmentation, the absence of evaluation on larger ViT models (e.g., ViT-Giant), and the lack of assessment in cytopathology and hematopathology domains. Moreover, its current design is unimodal and operates at the ROI level. Despite these challenges, UNI marks a significant step forward toward versatile and practical clinical applications in pathology [58].

#### CONCH

Building on this, the CONCH model (for CONtrastive learning from Captions for Histopathology) from the same lab employs a multimodal transformer that fuses image and text embeddings, trained on over 1.17 million image–caption pairs through task-agnostic pretraining. It exhibits state-of-the-art performance across 14 tasks in CPath, including image classification, captioning, tissue segmentation, and text-to-image as well as image-to-text retrieval [72, 73]. CONCH is regarded as a significant advancement over other existing visual-language pretrained systems for histopathology, with the capability to directly support a broad spectrum of ML workflows that require minimal or no additional supervised fine-tuning. Compared to widely used self-supervised encoders in CPath that were pretrained solely on H&E images, CONCH is likely to generate more effective representations for non-H&E-stained images such as IHC and special stains. Furthermore, it can be applied to a broad spectrum of downstream tasks involving either or both histopathology images and textual data. For example, clinicians can utilize textual queries such as “Breast cancer gene (BRCA)-mutated serous carcinoma” to identify morphologically similar cases, thereby significantly improving diagnostic accuracy.

#### GigaPath

In parallel, Microsoft’s GigaPath model leverages a LongNet transformer architecture to process gigapixel WSIs as sequential token streams [74]. Evaluation of GigaPath was conducted using a comprehensive digital pathology benchmark encompassing nine cancer subtyping tasks and 17 pathomics tasks, utilizing data from both Providence and TCGA. With large-scale pretraining and ultra-large-context modeling, GigaPath achieves state-of-the-art performance on 25 out of 26 tasks, including Lynch syndrome screening, significantly outperforming the second-best method on 18 tasks. Overall, GigaPath is an open-weight FM that attains leading performance across various digital pathology tasks, highlighting the critical importance of leveraging real-world data and whole-slide modeling. Its design emphasizes scalability and context preservation, enabling comprehensive analysis across large tissue sections [74, 75]. While GigaPath has achieved cutting-edge performance compared to previous leading models, there is still considerable room for growth across many downstream tasks. Although initial explorations into pathology vision-language pretraining have been promising, significant progress is needed to fully realize the potential of multimodal conversational assistants, particularly by integrating advanced multimodal frameworks such as Large Language and Vision Assistant for BioMedicine (LLaVA-Med) [76]. Most importantly, the influence of GigaPath and whole-slide pretraining on critical areas of precision health, such as modeling the tumour microenvironment (TME) and predicting treatment response, has yet to be thoroughly investigated.

#### mSTAR

Previous studies have primarily relied on either vision-only data or vision-caption pairs, overlooking valuable sources such as pathology reports and gene expression profiles, which provide complementary knowledge essential for versatile clinical applications. Therefore, the mSTAR model (for Multimodal Self-taught Pretraining), developed by Chen’s lab integrates WSIs with RNA sequencing data and clinical notes through a tri-modal transformer architecture [77]. Developed the most comprehensive oncological benchmark to date, covering seven categories of cancer-related applications across 15 types and a total of 97 practical oncological tasks. The experimental results demonstrate that mSTAR surpasses previous state-of-the-art FMs, especially excelling in molecular prediction, report-based pathology tasks, and multimodal fusion. This indicates that integrating additional pathology-related modalities at the slide level during pre-training greatly improves the model’s generalization capabilities for those specific modalities, thereby confirming the scalability of modality inclusion in the development of pathology FMs.

#### Atlas

Atlas is a novel FM for CPath developed through a collaboration between Mayo Clinic, Charité – Universitätsmedizin Berlin, and Aignostics in 2025 [78]. Based on the RudolfV training paradigm [79], it employs a ViT-H/14 with 632 million parameters, trained on an extensive and diverse dataset comprising 1.2 million histopathology WSIs from over 490,000 cases. This dataset includes a broad spectrum of disease, technical, and biological variability, encompassing more than 70 tissue and organ types and 100 staining types. In a comprehensive evaluation across 21 publicly available benchmark datasets, addressing a variety of morphological and molecular tasks, Atlas demonstrated state-of-the-art performance. It achieved the highest scores on 11 of these 21 tasks and attained an overall average performance of 61.9%. This represents a 1.1 percentage point improvement over the nearest competing models, despite not being the largest model in terms of parameters or training dataset size. These results suggest that Atlas offers a robust and generalizable foundation for a wide range of downstream applications in pathology [78].

### Clinical deployment and performance

#### Cancer diagnosis and subtyping

Foundation models have demonstrated significant promise in cancer diagnosis and subtyping, often achieving performance comparable to or exceeding that of specialized diagnostic techniques. They accomplish this through precise quantitative assessment of diverse tissue features, including immunohistochemical analysis, cell counting, spatial organization of cells, tissue density, distribution patterns, and overall tissue architecture [48]. For example, Paige AI Inc., in partnership with Microsoft Corp., introduced Virchow, a 632-million-parameter FM trained on 1.5 million H&E slides, enabling a single pan-cancer detector. This model achieved a specimen-level AUC of 0.95 across 16 cancers (nine common, seven rare) in a test set of 6,142 specimens, providing robust metrics that address the raised concern directly [80]. The model’s capacity to detect subtle morphological features linked to particular cancer subtypes marks a notable breakthrough in the field of CPath [81].

The effectiveness of cancer subtyping varies considerably among different tissue types and cancer subtypes. For common cancers such as breast, lung, and colorectal cancer, FMs consistently demonstrate high levels of diagnostic accuracy [79]. For example, when pathologists use tools such as Mindpeak Breast Ki-67 RoI and Mindpeak ER/PR RoI, which automate the assessment of Ki-67, estrogen, and progesterone receptors in breast cancer, their agreement improves. The statistical analysis indicates high interobserver variability among pathologists in conventional IHC quantification (Krippendorff's α, 0.69), with agreement increasing under AI support (Krippendorff α, 0.72) [82]. Conversely, performance in rare cancers and complex diagnostic situations continues to be an area of ongoing research and improvement [83].

#### Biomarker identification and prediction

Predicting molecular biomarkers directly from histological images is among the most promising applications of FMs. These models can detect morphological features associated with genetic mutations, protein expression, and other molecular traits without the need for costly molecular assays [84]. For example, Campanella et al. [85] assembled a large international clinical dataset comprising digital WSIs of lung adenocarcinoma (*n* = 8,461) to develop a computational biomarker for EGFR mutation status. By fine-tuning an open-source FM, their approach enhanced TS performance and demonstrated robust generalizability across external centers, achieving clinical-grade accuracy in both primary and metastatic specimens (mean AUC: internal 0.847, external 0.870). To assess real-world clinical applicability, the authors conducted a prospective silent trial on primary samples, where the model attained an AUC of 0.890. Integration of this AI-assisted workflow into clinical practice reduced the need for rapid molecular testing by up to 43%, while preserving established standards of diagnostic performance [85].

Beyond EGFR, FMs have shown promising efficacy in estimating a spectrum of clinically actionable biomarkers, including tumour mutation burden and other driver mutations, thereby directly informing personalized treatment strategies and potentially improving patient outcomes [48]. The intrinsic digital nature of these AI-driven computational biomarkers facilitates seamless integration into digital pathology workflows, enabling scalable, cost-effective, and highly accurate analyses. This capability not only augments precision oncology but also promotes the democratization of advanced diagnostics by allowing remote deployment, thereby expanding access to specialized care in underserved regions globally.

#### Survival prognostication and risk stratification

Prognostic modeling represents another critical application area where FMs excel. After years of research, various morphological features of histopathological tissues, such as tumour grade and tissue subtypes, have been established and proven to be useful indicators for predicting patient prognosis. By learning complex morphological patterns associated with patient outcomes, these models can provide valuable prognostic information that complements traditional clinical variables. The integration of multiple data modalities, including histological images, clinical variables, and molecular data, enables more accurate survival predictions. For instance, Wang et al. [49] elegantly demonstrated that the Clinical Histopathology Imaging Evaluation Foundation (CHIEF) model exhibits robust prognostic capabilities by stratifying cancer patients into distinct survival groups. It achieved an average concordance index of 0.74 for survival prediction, outperforming state-of-the-art methods by 7–12%. Validated across 17 independent cohorts (*n* = 9,404 WSIs), CHIEF significantly differentiated high- and low-risk patients (log-rank *P* < 0.05), providing a generalizable tool for mortality risk assessment. In addition, Shi et al. [86] developed a TME signature utilizing DL techniques applied to H&E-stained WSIs from a cohort of 562 CRC patients. This signature, which includes the stromal ratio and tumour-stroma ratio, proved to be a significant prognostic indicator for progression-free survival. When integrated with clinical variables, the model achieved a concordance index of 0.714, surpassing the predictive performance of individual TME features.

### Validation and community impact

Despite their promising potential, FMs face significant barriers to clinical adoption, even as they catalyze the development of new collaborative ecosystems. For example, a significant validation challenge is the performance gap when models are applied to unseen data without prior training [87]. A large-scale retrospective study directly compares FMs to a TS model for the diagnostic task of prostate cancer detection and Gleason grading from core needle biopsy WSIs. The findings challenge the assumption of universal FM superiority. While FMs demonstrated utility in data-scarce scenarios, their performance was highly dependent on extensive TS training [51]. For instance, with only 1% of the training data, the UNI model achieved approximately half the accuracy (as measured by the primary metric of quadratically weighted kappa, QWK, for the International Society of Urological Pathology (ISUP) grade) of the TS model on independent external validation cohorts. In some specific cohorts, its performance, indicated by negative QWK values, was even worse than random guessing [51]. However, when trained on 100% of the available data (~ 55,000 WSIs), the performance gap narrowed significantly across all models. Despite this, the end-to-end TS model maintained a minor but consistent advantage in Out-of-Distribution (OOD) generalization. The OOD criteria were rigorously defined as validation on cohorts from different patients, different clinical laboratories, and different WSI scanner models—completely independent of the development data. Furthermore, the study highlighted critical efficiency and sustainability concerns. Under a standardized energy measurement protocol (recording total kWh consumed by an NVIDIA A100 GPU to process a fixed set of 801 slides), the FMs were found to be vastly less efficient. The UNI and Virchow models consumed approximately 11 and 35 times more energy, respectively, than the TS model to perform the same diagnostic task. Overall, while FMs offer clear value for rapid prototyping, this evidence suggests that for well-defined clinical tasks with ample training data, a streamlined, end-to-end TS model can achieve superior or comparable generalization with a fraction of the computational cost and environmental impact [51].

Beyond validation, many clinicians distrust FMs due to their “black box” nature; a survey found that most of pathologists reject model outputs lacking visual explainability [88]. Nevertheless, community-driven advancements are helping to address some of these challenges. The Hugging Face ecosystem, with over 1.5 million downloads of pathology FMs, has enabled tasks such as grading and subtyping neural tumours called neuroblastomas, predicting treatment outcomes, and identifying gene-expression biomarkers associated with specific diseases [89].

## Emerging applications: AI “copilots” and beyond

**Fig. 3:**
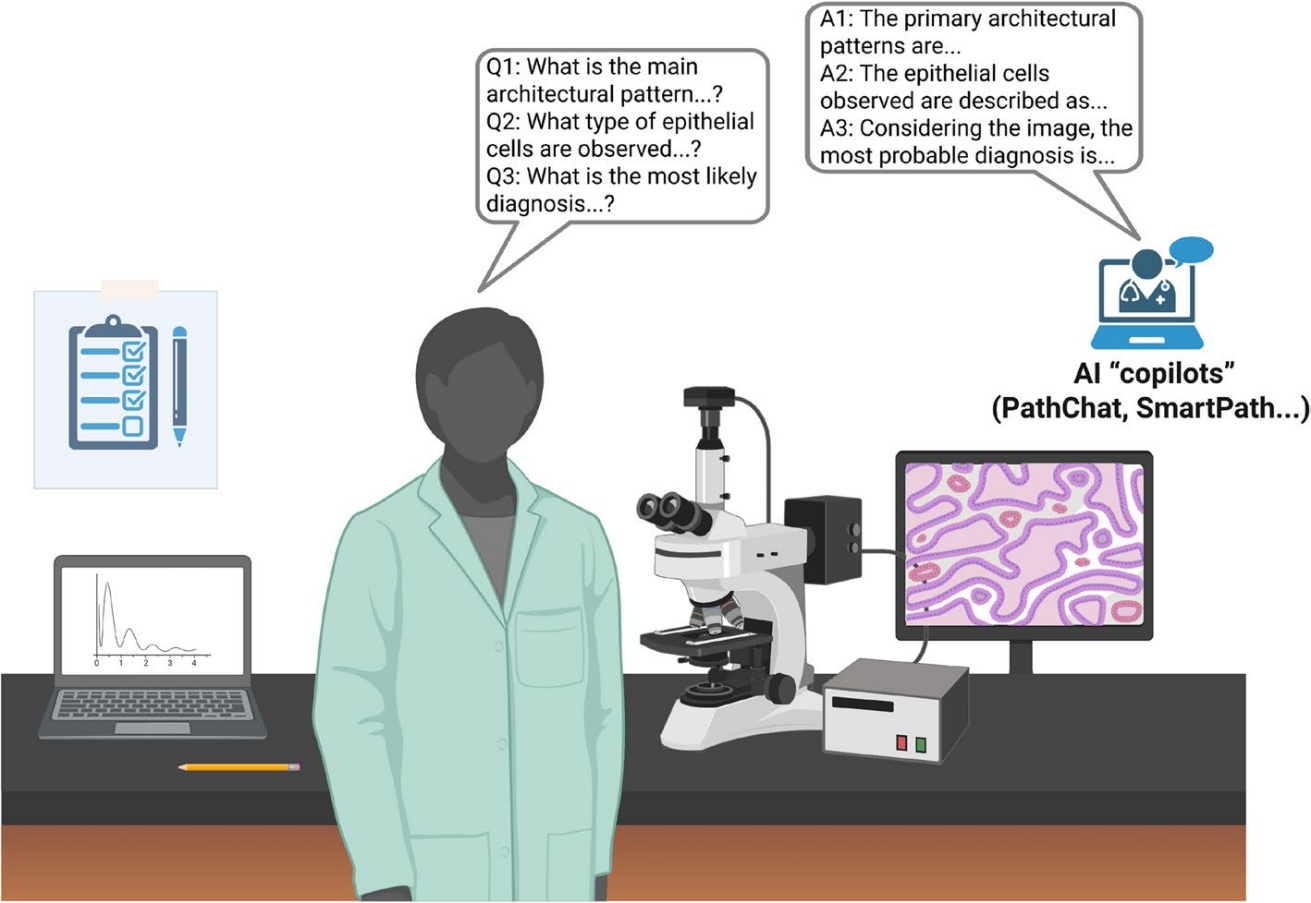
Simulates how AI "copilots" will assist pathologists in diagnosing and evaluating diseases

Recent multimodal AI assistants such as PathChat, PathAsst, SmartPath, and SlideChat are transforming pathology practice. These systems seamlessly integrate visual and textual data to support complex tasks, including diagnostic reasoning, morphological feature description, biomarker testing recommendations, and prognostic assessment. Their development marks a paradigm shift toward collaborative AI "copilots" that enhance pathologist workflows in precision diagnostics and data interpretation (Fig. 3).

### PathChat

In 2024, Mahmood’s team [90] released PathChat, a generalist AI assistant that integrates UNI with large language models (LLMs) to deliver versatile and comprehensive support. Specifically, it has been assessed using multiple-choice diagnostic questions encompassing 54 different diagnoses across 11 major pathology practices and organ sites. PathChat achieved a diagnostic accuracy of 78.1% based on images alone, which significantly improved to 89.5% when additional clinical context was provided. It notably outperformed other state-of-the-art models, including GPT-4 V (90.5% vs. 63.5% accuracy with clinical context). Additionally, open-ended questions were employed to evaluate its performance on a wide range of topics, including microscopy image description, histologic grading and differentiation status, risk factors, prognostic assessments, treatment options, diagnostic processes, IHC testing, molecular alterations, and other diagnostic tests. In expert evaluations, PathChat's responses were preferred over those from GPT-4 V in 56.5% of cases and achieved an overall accuracy of 78.7% on consensus questions, surpassing GPT-4 V by 26.4% [90]. To evaluate PathChat, the researchers curated PathQABench by selecting representative high-resolution ROI from 105 H&E WSIs, using the open-source QuPath digital viewer, with hand-selected annotations provided by a board-certified pathologist [90, 91]. It demonstrated its ability to summarize key morphological features in histology microscopy images, formulate diagnoses by integrating histological and clinical contexts, assess tumour grade and differentiation, recommend additional IHC and molecular testing, and provide insights into risk factors, prognosis, and therapeutic options for the underlying disease.

Overall, PathChat generated more accurate and pathologist-preferred responses to a wide array of queries related to pathology. As an interactive vision-language AI assistant capable of seamlessly handling both visual and natural language inputs, PathChat holds significant potential for impactful applications in pathology education, research, and human-in-the-loop clinical decision-making. Pathologists can utilize PathChat to engage in interactive discussions about uploaded images and to generate comprehensive reports, among other functionalities. Building on these findings, PathChat, licensed to Modella AI, a biomedical company based in Boston, Massachusetts, received breakthrough device designation from the U.S. food and drug administration (FDA) earlier this year [90, 92, 93]. Its potential applications are broad, encompassing pathology education, research, and human-in-the-loop clinical decision-making, with the promise of increasing impact as the underlying technology continues to evolve and mature.

### PathAsst

PathAsst, another multimodal generative foundational AI assistant, was designed to transform diagnostic and predictive analytics in pathology [94]. The development of PathAsst encompasses three key stages: data acquisition, adaptation of the contrastive language-image pretraining (CLIP) model, and the training of its multimodal generative capabilities. Over 207,000 high-quality pathology image-text pairs were curated from authoritative sources such as PubMed and pathology books to create the PathCap dataset, and specialized instruction data was devised by generating over 180,000 instruction-following samples via ChatGPT to create the PathInstruct dataset, which was tailored to invoke eight pathology-specific sub-models. This setup enables PathAsst to collaborate effectively with these sub-models, thereby enhancing its diagnostic capabilities. Using the collected data, researchers subsequently constructed PathCLIP, a pathology-specific CLIP model, which demonstrated a remarkable 11.07-fold and 10.71-fold improvement in R@10 image retrieval on PubMed data compared to previous state-of-the-art models PLIP and OpenAI CLIP, respectively, thereby substantially improving the system’s ability to interpret pathology images. PathCLIP was integrated with Vicuna-13b and further refined through pathology-specific instruction-tuning data, which augmented PathAsst’s multimodal generative capacity and reinforced its synergistic interactions with the sub-models. In evaluations on the PathVQA dataset, PathAsst achieved a 90.9% accuracy on closed-ended questions and a 38.4% F1-score on open-ended questions, outperforming existing multimodal models. Experimental results demonstrate that PathAsst holds substantial promise in leveraging AI-powered FMs to advance pathology diagnosis and treatment.

### SmartPath

SmartPath, introduced by Chen’s team, is a versatile pathology co-pilot capable of concurrently addressing both ROI-level and WSI-level tasks across diverse anatomical regions, all while demonstrating robust capabilities in pathological reasoning [95]. This framework integrates scale-dependent supervised fine-tuning with task-aware reinforcement fine-tuning, effectively circumventing the need for chain-of-thought supervision by leveraging the inherent knowledge embedded within multi-modal large language models (MLLMs). Additionally, SmartPath employs a mixture-of-experts mechanism to facilitate multiscale and multitask analysis, enabling dynamic and adaptable processing across diverse tasks. To support training and evaluation, a large-scale dataset was curated, comprising 2.3 million ROI samples and 188,000 WSI samples. Extensive experiments spanning 72 tasks demonstrate the effectiveness and superiority of the proposed approach. Quantitative evidence from these experiments solidifies its state-of-the-art performance: across the 72 tasks, SmartPath-R1 achieved a top average rank score of 1.1, ranking first in 68 of them. It significantly outperformed existing models in key areas, achieving a 38.3% higher average accuracy in ROI-level classification and a 15.5% absolute margin in accuracy for WSI-level classification. Overall, SmartPath marks a significant advancement toward the development of versatile, reasoning-enhanced AI systems tailored for precision pathology.

### SlideChat

SlideChat is a vision-language assistant capable of understanding gigapixel WSIs, demonstrating exceptional multimodal conversational capabilities and the ability to respond to complex instructions across a wide range of pathology scenarios [96]. To facilitate its development, SlideInstruction was created, encompassing 4,181 WSI captions and 175,753 visual question-answering (VQA) pairs from 3,294 patients across ten cancer types, which is more than 20 times larger than previous public instruction datasets. Furthermore, researchers introduced SlideBench, a multimodal benchmark integrating captioning and VQA tasks to evaluate SlideChat’s performance across diverse clinical contexts, comprising 734 WSI-caption pairs, 7,827 VQA pairs from TCGA, and 7,274 VQA pairs from an external BCNB dataset. Compared to both general and specialized MLLMs, SlideChat demonstrates exceptional capabilities, achieving state-of-the-art results on 18 out of 22 tasks. For instance, it attained an overall accuracy of 81.17% on SlideBench-VQA (TCGA), representing a significant average accuracy improvement of 13.47% over the second-best model, and 54.15% on SlideBench-VQA (BCNB), which is an average improvement of 12.59%. The model also showed strong generalization, achieving a 5.82% improvement on the external WSI-VQA benchmark.

## Critical challenges of AI in cancer pathology

### Data heterogeneity and standardization barriers

The primary technical challenge stems from the inherent complexity of WSIs, which are vast gigapixel visuals exhibiting considerable variability owing to differences in sample preparation and scanning techniques. This heterogeneity poses significant obstacles to developing models capable of generalizing across different institutions, a crucial requirement for expanding the widespread impact of AI in pathology [23]. Further complicating the situation are concerns regarding patient privacy and data security. Annotating gigapixel images continues to be a resource-intensive process, and the sharing of multi-center datasets is hindered by privacy restrictions. Although regulations such as the health insurance portability and accountability act (HIPAA) and general data protection regulation (GDPR) are designed to safeguard patient information, they pose significant obstacles to data sharing across institutions, thereby impeding collaborative efforts in AI development [97].

Another critical hurdle in the adoption of AI in pathology is the lack of standardization, particularly concerning the interoperability between different systems [98]. Studies highlight the crucial role of LLMs in advancing data standardization efforts [99, 100]. For example, the CORAL dataset, which incorporates 9,028 expert-annotated entities and 5,312 relationships from 40 oncology progress notes, has demonstrated significant improvements in extracting complex oncological information. Fine-tuning LLMs on this high-quality, structured data enabled GPT-4 to achieve an average BLEU score of 0.73, a ROUGE score of 0.72, and an expert-evaluated accuracy of 68% in zero-shot inference tasks, with particularly strong performance in tumour characteristic extraction (e.g., BLEU score of 0.95 for tumour grade) and medication history synthesis [101]. Without established standards, AI-generated results might be difficult to interpret across various platforms or might necessitate manual adjustments, thereby compromising efficiency. Standardizing AI outputs is essential to facilitate seamless integration with other software tools commonly employed in pathology laboratories, ultimately streamlining the entire workflow [102]. By automating routine tasks and delivering precise, timely information to pathologists, AI can transform digital workflows into more efficient alternatives to traditional manual methods, leading to faster diagnoses and better clinical outcomes. Therefore, establishing standardized AI results is not merely a technical requirement but a critical step toward unlocking the full capabilities of AI in pathology.

### Model reliability and clinical trust gaps

AI models, particularly DL architectures, are often described as “black boxes” due to their inherent difficulty in providing clear interpretations [103]. Essentially, data is processed to generate an output, yet the underlying mechanisms remain opaque, resembling a black box. This lack of transparency can present significant challenges across diverse fields, particularly in ensuring fairness, accuracy, and accountability. In healthcare settings, where accountability and transparency are paramount, this lack of interpretability presents a significant obstacle to clinical adoption [104]. Methods such as layer-wise relevance propagation (LRP) and shapley additive explanations (SHAP) are being actively developed to enhance the interpretability of AI models by identifying the most influential components of the input data in the model’s predictions [105]. Regulatory agencies such as the FDA also mandate that AI systems be explainable. Despite these advancements, attaining complete transparency continues to be a significant challenge in AI-driven cancer pathology [106, 107].

Clinical trust gaps in AI-powered pathology stem from challenges in ensuring AI's reliability, transparency, and fairness, particularly in the context of complex diagnostic tasks and potential biases in training data [108]. AI algorithms are trained on data, and if that data reflects existing biases in healthcare, the AI can perpetuate or even amplify those biases, leading to inaccurate diagnoses or unfair outcomes for certain patient populations. For example, studies have shown performance gaps in AI systems when applied to different racial groups [109, 110].

### Workflow integration and resource constraints

While AI has the potential to transform cancer pathology, it is important to emphasize that AI is intended to complement, not replace, human pathologists. AI systems can analyze large volumes of data rapidly and consistently, but pathologists provide essential contextual understanding and clinical judgment that AI cannot replicate. The key challenge is integrating AI into the clinical workflow in a way that improves both efficiency and accuracy without undermining the role of human pathologists. Creating user-friendly AI tools that pathologists can trust and work collaboratively with is crucial for the successful adoption of this technology [111, 112].

Another hurdle involves the need for a thorough assessment of infrastructure costs and energy consumption. For example, hospitals and laboratories need to invest in new hardware and software, including digital scanners, to support AI-powered pathology [113]. Processing gigapixel-sized WSIs requires substantial computational resources, and not all institutions have access to the high-performance computing infrastructure needed for AI analysis. Although cloud computing offers some relief, achieving real-time, low-latency analysis remains a major hurdle, as it is essential for timely clinical decision-making [114]. Furthermore, the scalability of AI-powered pathology solutions is limited by hardware constraints and the energy demands of AI models. Deploying AI across multiple institutions, especially in resource-limited settings, poses significant challenges. A comprehensive assessment of hardware costs, limitations, and energy consumption is essential to understand their influence on research and development expenses, as well as the barriers to adoption in the context of FM development [4].

### Ethical governance and risk of misapplication

The interaction between AI and pathologists brings forth important ethical questions. Although AI has the potential to minimize human error and enhance diagnostic efficiency, it should serve as a complement to human expertise. Ethical guidelines should emphasize that AI is an augmentation tool intended to assist pathologists in making more informed decisions, while maintaining the essential role of human judgment, particularly in complex or ambiguous cases [115]. As AI systems assume greater autonomy in diagnostic decision-making, another question of accountability emerges: who is responsible when an AI system makes an error? Addressing these ethical and societal issues is crucial for building trust in AI technologies and ensuring their fair and responsible deployment in clinical practice [116].

Over-reliance on AI systems presents another potential risk, as pathologists might depend too heavily on AI suggestions, thereby diminishing human oversight and critical thinking [117]. This issue could be especially problematic in complex or rare cases where AI models might not have enough training data to provide accurate predictions. Nevertheless, strategically deployed FMs can mitigate these risks while democratizing advanced AI capabilities, fostering innovation, and enhancing outcomes in precision medicine and pathology research. By expanding the availability of these models and actively addressing concerns related to bias and accountability, the healthcare industry can work toward more equitable healthcare solutions.

## Future directions and limitations

The integration of AI, especially FMs and AI “copilots”, heralds a transformative era in cancer pathology. These technologies address critical challenges such as workforce shortages, diagnostic complexity, and workflow inefficiencies by enhancing precision, scalability, and data integration. To unlock their full clinical potential, targeted advancements are essential across technical, clinical, and ethical domains. On the technical front, future development should prioritize robust multimodal integration that extends beyond histology-text pairs to include genomics, radiomics, and real-time clinical data streams, exemplified by initiatives such as mSTAR. Additionally, scaling gigapixel modeling techniques to facilitate comprehensive TME analysis, while optimizing computational efficiency, is vital. Equally important is the development of trustworthy AI frameworks that incorporate interpretability techniques to clarify decision-making processes, particularly in high-stakes diagnostic scenarios. Ensuring the models' generalizability across diverse institutions involves mitigating performance variability through stain normalization, federated learning, and synthetic data augmentation.

Clinically, the focus should be on designing AI tools that seamlessly integrate into existing workflows, promoting effective human-AI collaboration rather than mere automation. Tools such as SmartPath and SlideChat should be embedded into pathologist routines to enhance diagnostic efficiency and accuracy. Establishing multi-institutional validation benchmarks, such as extensions of PathQABench, is crucial for evaluating robustness across diverse populations and rare cancer types. Additionally, developing lightweight, resource-efficient model variants can help democratize access, particularly in low-resource settings, ensuring broader clinical adoption.

Ethical and regulatory considerations are equally paramount. Efforts must concentrate on bias mitigation by auditing training datasets to address representation gaps and on establishing clear accountability standards for diagnostic errors involving AI “copilots” such as PathAsst’s sub-models. Close collaboration with regulatory agencies such as the FDA and EMA is essential to streamline approval processes for adaptive AI systems. Initiatives such as the FDA’s designation of PathChat exemplify how regulatory frameworks can evolve to facilitate the safe and effective deployment of innovative AI tools, ultimately translating technological advancements into reliable clinical solutions.

This review has several limitations that warrant acknowledgment. Firstly, while we have striven to provide a comprehensive overview of the rapidly evolving landscape of AI in pathology, the field is advancing at an extraordinary pace. Consequently, some of the most recent developments, particularly concerning the clinical deployment and real-world performance of the latest foundation models and copilots, might not be fully captured by the time of publication. Secondly, our analysis and the conclusions drawn are inherently constrained by the scope and quality of the existing primary literature. Many studies, especially those introducing novel foundation models, are still in the pre-print stage or have been validated on retrospective datasets, which may not fully represent the complexities and heterogeneities of prospective, multi-institutional clinical practice. This introduces a potential publication bias, where positive results are overrepresented. Furthermore, the performance metrics reported across various studies exhibit considerable variability attributable to differences in datasets, benchmarking methodologies, and evaluation criteria. This variability renders direct and definitive comparisons between models challenging. A substantial portion of the discourse concerning challenges—such as data standardization, algorithmic bias, and regulatory obstacles—is predominantly founded on theoretical considerations and preliminary empirical evidence, as large-scale, longitudinal investigations evaluating the long-term effects and safety of these integrated AI systems remain in the early stages of development. Addressing these limitations will require concerted efforts in robust multi-center trials, the development of standardized benchmarks, and proactive engagement with regulatory bodies to translate the promising potential of FMs and copilots into trustworthy, equitable, and clinically impactful tools.

## Conclusion

Artificial intelligence-based foundation models and “copilots” are redefining pathology’s role in precision oncology. From UNI’s few-shot disease classification to GigaPath’s whole-slide modeling and PathChat’s diagnostic reasoning, these tools unlock unprecedented capabilities in biomarker discovery, rare cancer diagnosis, and prognostic modeling. Yet their clinical impact hinges on resolving critical challenges: data heterogeneity, model transparency, and equitable access. By advancing multimodal integration, fostering human-AI collaboration, and establishing ethical guardrails, the field can transition from fragmented TS tools to unified, trustworthy AI ecosystems. As these technologies mature, they promise not merely to augment pathologists but to catalyze a new paradigm—where CPath accelerates personalized therapeutics, democratizes diagnostic expertise, and ultimately elevates patient care globally.

## Data Availability

No datasets were created or analysed in this study.

## Abbreviations

AI: Artificial intelligence
AUC: Area under the curve
BC: Breast cancer
CAMELYON: CAncer MEtastases in LYmph nOdes challeNge
CHIEF: Clinical Histopathology Imaging Evaluation Foundation
CLIP: Contrastive Language-Image Pretraining
CNN: Convolutional neural network
CONCH: CONtrastive learning from Captions for Histopathology
CPath: Computational pathology
CRC: Colorectal cancer
DCIS: Ductal carcinoma in situ
DL: Deep learning
EGFR: Epidermal growth factor receptor
EMA: European Medicines Agency
FDA: U.S. Food and Drug Administration
FM: Foundation model
GAN: Generative Adversarial Network
GDPR: General Data Protection Regulation
GPU: Graphics processing unit
H&E: Hematoxylin and eosin staining
HER2: Human epidermal growth factor receptor 2
HIPAA: Health Insurance Portability and Accountability Act
IHC: Immunohistochemistry
ISUP: International Society of Urological Pathology
JSCCR: Japanese Society for Cancer of the Colon and Rectum
LLaVA-Med: Large Language and Vision Assistant for BioMedicine
LLMs: Large language models
LRP: Layer-wise Relevance Propagation
LUAD: Lung adenocarcinoma
LUSC: Lung squamous cell carcinoma
ML: Machine learning
MLLMs: Multi-modal large language models
MSI: Microsatellite instability
mSTAR: Multimodal Self-taught Pretraining
NSCLC: Non-small-cell lung cancer
OOD: Out-of-Distribution
PD-L1: Programmed cell death ligand 1
QWK: Quadratically Weighted Kappa
ResNet: Residual network
ROI: Regions of interest
SCLC: Small cell lung carcinoma
SHAP: SHapley Additive exPlanations
SSL: Self-supervised learning
TCGA: The Cancer Genome Atlas
TME: Tumour microenvironment
TS: Task-specific
ViT: Vision Transformer
VIS: Visiopharm Integrator System
VQA: Visual Question-Answering
WSI: Whole slide imaging
